# Composition of gut microbiota in infants in China and global comparison

**DOI:** 10.1038/srep36666

**Published:** 2016-11-09

**Authors:** Ya-Shu Kuang, Sheng-Hui Li, Yong Guo, Jin-Hua Lu, Jian-Rong He, Bei-Jun Luo, Feng-Ju Jiang, Hui Shen, Christopher J. Papasian, Herbert Pang, Hui-Min Xia, Hong-Wen Deng, Xiu Qiu

**Affiliations:** 1 Division of Birth Cohort Study, Guangzhou Women and Children’s Medical Center, Guangzhou Medical University, Guangzhou 510623, People’s Republic of China; 2 Department of Obstetrics, Guangzhou Women and Children’s Medical Center, Guangzhou Medical University, Guangzhou 510623, People’s Republic of China; 3 Center of Bioinformatics and Genomics, Department of Biostatistics and Bioinformatics, Tulane School of Public Health and Tropic Medicine, New Orleans, Louisiana, USA; 4 Department of Basic Medical Science, School of Medicine, University of Missouri – Kansas City, Kansas City, Missouri, USA; 5 School of Public Health, Li Ka Shing Faculty of Medicine, Hong Kong, China; 6 Department of Neonatal Surgery, Guangzhou Women and Children’s Medical Center, Guangzhou Medical University, Guangzhou 510623, China; 7 Department of Woman and Child Health, Guangzhou Women and Children’s Medical Center, Guangzhou Medical University, Guangzhou 510623, China

## Abstract

Symbiotic gut microbiota is essential for human health, and its compositional changes have been associated with various complex disorders. However, systematic investigation of the acquisition and development of gut microbial communities during early infancy are relatively rare, particularly for infants from non-Western countries. In this study, we characterize the colonization and development of infant microbiota in healthy Chinese infants and compare the pattern with those from other countries. The fecal microbiota of 2-month-old infants was considerably more diverse than that of neonates, as indicated by higher relative abundances of *Veillonella*, *Clostridium*, *Bacteroides*, *Lactobacillus*, *Collinsella* and *Prevotella*, and reduction of *Escherichia* and *Enterococcus*. The fecal microbiota of vaginally delivered infants (both neonates and 2-month-old) had significant enrichment of *Bacteroides*, *Parabacteroides* and *Megamonas*, whereas cesarean delivered infants had enrichment of *Prevotella*, *Streptococcus* and *Trabulsiella*. By global comparison, we identify three different enterotypes, referred as “P-type”, “A-type ”and “F-type” which were highly abundant in Proteobacteria, Actinobacteria and Firmicutes, respectively. The three enterotypes’ compositons vary geographically. All Chinese infants in our study belong to the P-type. These findings may provide novel insights into our understanding of the establishment of infant fecal bacterial communities.

Gut microbiota plays an important role in human health and disease development through a variety of different mechanisms including nutrient processing, protection against pathogens, stimulation of angiogenesis, regulation of host fat storage, and alterations in host behavior^1^,^2^,^3^. In infants, early acquisition of gut microbiota has been linked to development of innate immune responses and terminal differentiation of intestinal structures^1^,^4^. Studies on the initial colonization of the intestinal tract of infants have opened a window into our understanding of the succession dynamics of the gut microflora, and provided useful clues for the etiology of various complex diseases in adolescence and adulthood^1^,^5^,^6^,^7^,^8^,^9^. For instance, allergic children showed a delay in development of gut microflora^10^.

Different social structures may influence the extent of vertical transmission of microbiota from mother to infant, and the flow of microbes and microbial genes among members of a household. For example, different cultural influences such as exposure to pets and livestock could influence how and from where the gut microbiota/microbiome is acquired. It is essential to sample a broad population of healthy humans over time, both in terms of their age, geography and cultural traditions, to discover features of gut microbiomes that are unique to, and influenced by, different locations and lifestyles. A number of studies have characterized the colonization and development of infant microbiota in western countries^11^,^12^,^13^; however, little is known about this in healthy Chinese infants. Liu D *et al.*^14^ used 16S rDNA PCR-DGGE methodology to provide a profile of the dominant gut microbiota of Chinese neonates, but the PCR-DGGE method used may limit the generalizability of their results. It has been previously demonstrated that species richness and diversity were higher for the samples analyzed by 454-pyrosequencing than DGGE^15^.

Here we performed our study of the gut microbiota in healthy Chinese infants by adopting a more accurate technology (16S rDNA sequencing). We examined fecal samples from 15 full-term neonates during the first four days of life and 14 different full-term infants at approximately 1–3 months of age. We also assessed the impact of mode of delivery on the establishment of infant gut microbiota, and in particular, we compared the gut microbiota composition among infants from different countries, with the aim of identifying geographical patterns and influential factors.

## Materials and Methods

### Study population and fecal sample collection

Twenty-nine Chinese Han healthy babies, including 15 neonates aged 1–4 days and 14 infants aged 1–3 months, were recruited at the Guangzhou Women and Children’s Medical Center (GWCMC) as part of the Born in Guangzhou Cohort Study (BIGCS). Subjects were excluded if they had symptoms of respiratory infection or digestive tract disease, or had a history of treatment with antibiotics or anti-inflammatory agents. Mothers who had severe obstetric complications such as gestational diabetes mellitus, pregnancy-induced hypertension, preeclampsia or eclampsia were also excluded. Fecal samples were collected in sterile flask containers by perianal stimulation. To minimize the effect of experimental and sequencing error, each fecal sample was partitioned into 3 parallel samples and replicated in triplicate. Samples were stored at room temperature for no more than 30 minutes before freezing at −80 °C.

### Ethics Statement

This study received approval from the ethics committee of GWCMC, and written informed consent was obtained from parents of each child. The methods were carried out in accordance with the approved guidelines.

### Sample processing and 16S RNA sequencing

Total bacterial DNA was extracted from all samples using a previously reported method^16^. The V3–V5 hypervariable region of the 16S rRNA gene was amplified, sequenced and analyzed to define composition of the bacterial community. Amplification primers were designed with FLX Titanium adapters, primer-F: 5′CCATCTCATCCCTGCGTGTCTCCGACTCAG 3′; primer-R: 5′CCTATCCCCTGTGTGCCTTGGCAGTCTCAG 3′. For each sample, the 16S rRNA gene was amplified under the following conditions: initial denaturation at 94 °C for 3 min, followed by 30 cycles of 94 °C for 45 s, 56 °C for 1 min, and 72 °C for 1 min, followed by a final extension at 72 °C for 10 min. PCR products were quantified via gel electrophoresis, pooled and purified for sequencing reactions. Pyrosequencing was performed on a 454 GS FLX Titanium sequencer (454 Life Sciences, USA) at the genomics research institute BGI-Shenzhen, China.

### Data analysis

Low-quality sequencing reads were eliminated by using the PyroNoise algorithm in Mothur^17^ based on the following criteria: a) raw reads shorter than 400 bp; b) a sequence producing more than 8 homopolymers; c) over 2 mismatches in the primer, or d) one or more mismatches in the barcode.

Microbiome analysis was then implemented using the Quantitative Insights Into Microbial Ecology (QIIME) platform^18^. Briefly, 16S rRNA operational taxonomic units (OTUs) were clustered using an open-reference OTU picking protocol based on 97% nucleotide similarity with the UCLUST algorithm^19^. ChimeraSlayer was employed to remove chimeric sequences^20^. The most abundant sequence from each OTU was selected to determine the phylogeny of the OTU based on taxonomic classifier, RDP-classifier^21^. Taxonomic relative abundance profiles at taxa levels (phylum, class, order, family and genus) were generated based on OTU annotation. The between-sample diversity (beta diversity) was evaluated by weighted and unweighted UniFrac distances and calculated by the QIIME pipeline.

### Data for comparing gut microbiota of infants from different countries

To compare the gut microbiota composition among infants from different countries, we searched published articles for suitable infant microbiome data using the following inclusion criteria: 1) original research papers reporting independent data; 2) using 16S rDNA sequencing; 3) performed in healthy infants aged 1–4 months; 4) sample size no less than 10; and 5) original data could be downloaded. A Pubmed search of “microbiota” or “bacteria” and “16S” returned 485 unique articles, of which five studies conducted in five different countries met all predefined inclusion criteria and were thus included in our data analyses. Fourteen infants aged 1–3 months from our study were included to compare with infants from the other five studies (aged 1–4 months).

Because the included studies utilized some different technical approaches to generate data, microbiome analysis was implemented using the QIIME platform for each country (dataset) separately. Taxonomic relative abundance profiles at the phylum level were generated based on OTU annotation for each country separately. Principal component analyses (PCA) were then done based on the profiles obtained from various countries.

## Results

### Study population

Basic characteristics of the 29 healthy Chinese infants included in the study are summarized in Table 1. The study population consisted of 15 neonates with a mean age of 2.3 days and 14 infants with a mean age of 54.3 days. There were about equal numbers of boys and girls in each group, and 6 neonates (40%) and 7 infants (50%) were born by cesarean delivery.

### Overall phylogenetic profiles of gut microbiota of healthy Chinese infants

A total of 404,655 sequencing reads were generated and clustered into 6,529 OTUs. Of those, 55.2% of the OTUs were classified into genus-level taxa, accounting for 67.9% of the total reads. Almost all (96.5%) of the OTUs could be classified into the level of specific families.

At the phylum level, *Proteobacteria* (mean relative abundance = 52.3%), *Firmicutes* (40.2%), *Bacteroidetes* (6.1%) and *Actinobacteria* (0.9%) comprised the vast majority of the Chinese infant gut microbiota. At the family level, the gut microbiota was generally dominated by *Enterobacteriaceae* (mean relative abundance = 51.8%, with representation mainly by the genera *Escherichia/Shigella* and *Klebsiella*), *Enterococcaceae* (15.1%), *Streptococcaceae* (5.8%), *Lactobacillaceae* (5.5%), *Bacteroidaceae* (3.5%) and diverse families belonging to the phylum *Firmicutes*.

### Development of infant gut microbiota

**Figure 1:**
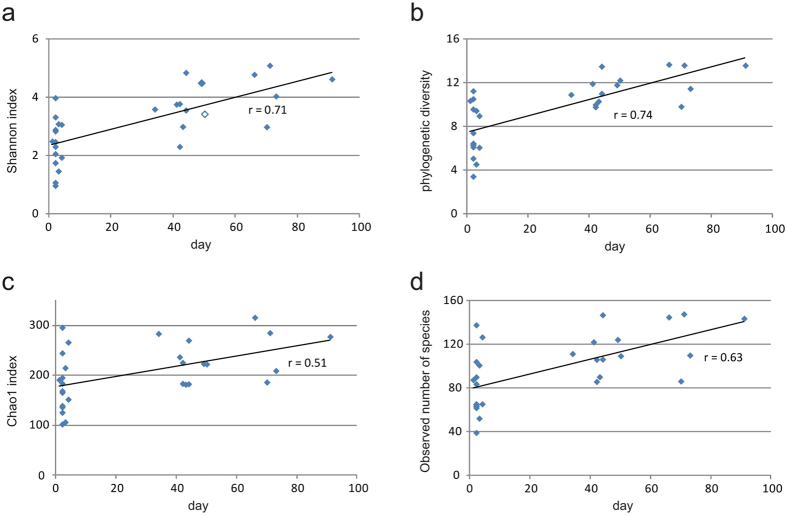
Alpha diversity indices as indicators of microbial community biodiversity of neonates and 2-month-old infants. Four estimators of alpha diversity are shown: (**a**) Shannon index, (**b**) phylogenetic diversity, (**c**) Chao1 index, and (**d**) observed number of species. For each estimator, values for each individual are plotted by points.

**Figure 2:**
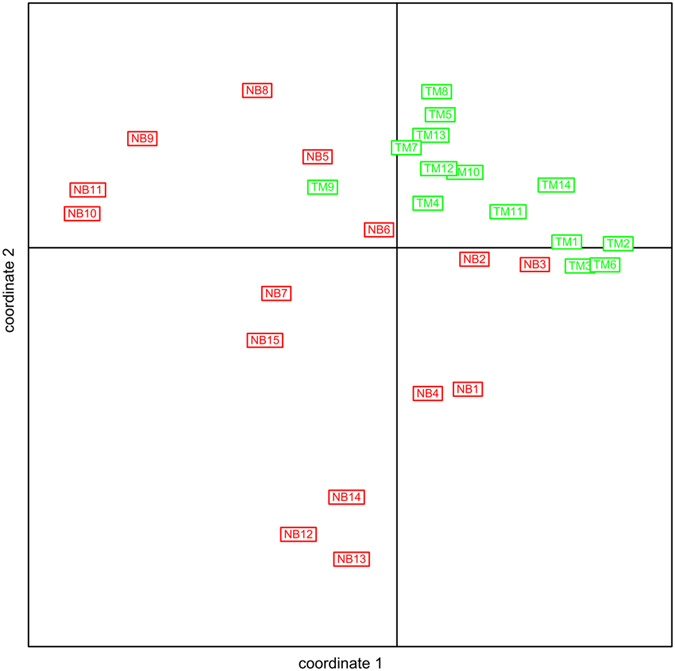
PCoA of the unweighted UniFrac distance as a measure of microbial community structure. The x- and y-axis indicate the first two coordinates.

We carried out biodiversity analyses by comparing the richness and evenness of the fecal microbial communities between neonates and ~2-month-old infants. Richness is merely a measure of the number of species in each sample, without taking into account the relative abundance of each species. Evenness, in contrast, takes into account both the number of species, and the relative abundance of each species. Within-sample (alpha) diversity was assessed by four indices: Shannon index, phylogenetic diversity, Chao1 index, and observed number of species. These indices estimated the richness and evenness of the microbial community of the fecal microbiome. All four of these indices were significantly lower in neonates than in 2-month-old infants (*p* < 0.01 for all comparisons, Student’s t test). Specifically, all four indices increased along with infant age, with correlation coefficients between postnatal days and within-sample diversity indices ranging from 0.51 to 0.74 (*p* < 0.01 for all indices, Pearson coefficients test) (Fig. 1). We used the UniFrac distance to measure the changes of community structure between these samples. Neonates and 2-month-old infants were clearly separated based on principal coordinate analysis (PCoA) of both weighted and unweighted UniFrac distances (Fig. 2), indicating significant compositional differences between the neonates and the 2-month-old infants.

**Figure 3:**
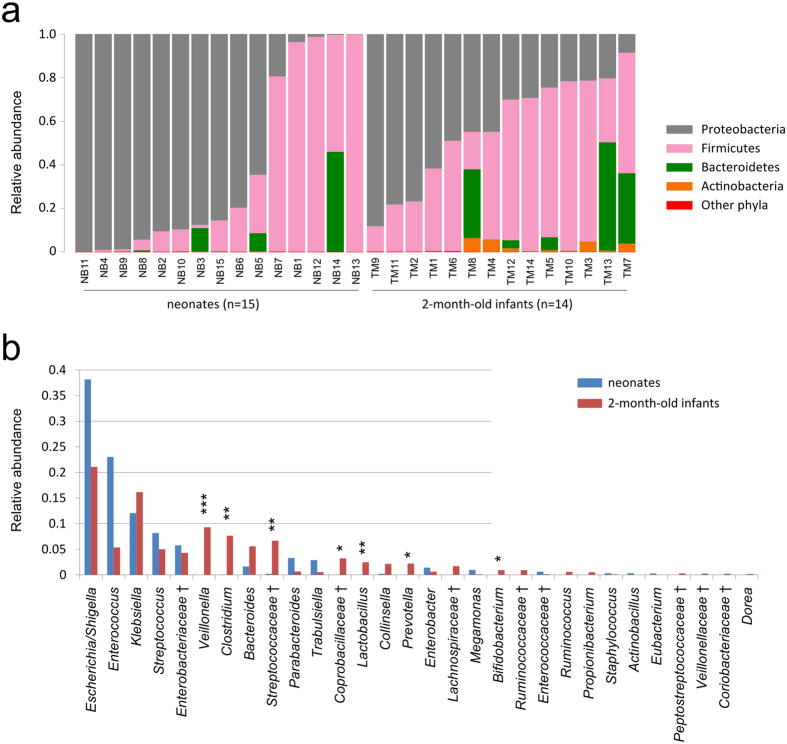
Comparison of microbial community composition at the phylum (**a**) and genus (**b**) levels for neonates and 2-month-old infants. Only the top 30 most abundant genera are shown for demonstration and clarity. Genera with significant differences between neonates and 2-month-old infants are marked by asterisks: “*” denotes *P* < 0.05; “**” denotes *P* < 0.01; “***” denotes *P* < 0.001. Unclassified genera under a higher rank are marked by “^†^”.

At the phylum level, compared with the neonates, the 2-month-old infants had slight decreases in abundance of *Proteobacteria* (43% vs. 61%), and increases in *Firmicutes* (47% vs. 35%) and *Bacteroidetes* (8% vs. 4%), though these changes were not significant (*p* > 0.05 for three phyla, Student’s t test). Another dominant phylum, *Actinobacteria*, was almost undetectable in the gut microbiota of neonates but was significantly enriched in the gut microbiota of 2-month-old infants (*p* = 0.02, with an average relative abundance of 2%, Fig. 3a). At family and genus levels, the gut microbiota of the 2-month-old infants was significantly enriched for *Veillonella* (*p* = 0.0003, Student’s t test), *Clostridium* (*p* = 0.0042), *Streptococcaceae* (*p* = 0.0091), *Coprobacillaceae* (*p* = 0.021), and *Lactobacillus* (*p* = 0.0037) among others, whereas the gut microbiota of neonates tended to have a higher abundance of *Escherichia/Shigella*, *Enterococcus*, *Parabacteroides* and *Trabulsiella* though these differences were not statistically significant (Fig. 3b).

### Effects of delivery modes on infant gut microbiota

**Figure 4:**
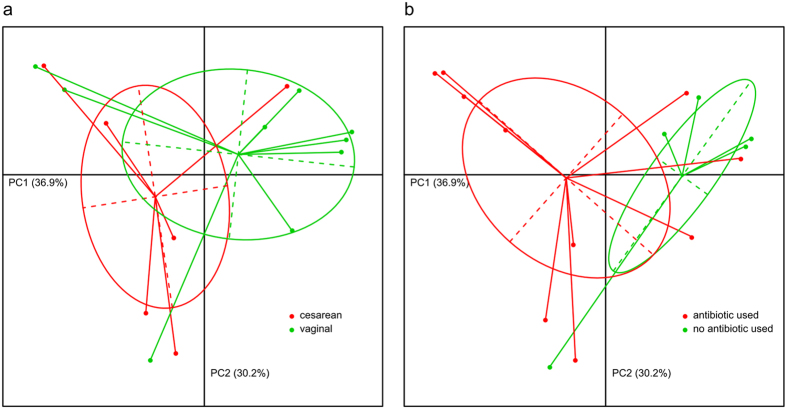
Principal component analysis of the genus profiles in neonatal gut microbiota. (**a**) Individuals are grouped by their delivery mode; (**b**) Individuals are grouped by the antibiotic using mode of their mother. The first two principle components (PCs) and their ratio of variance contribution are shown. Nodes represent the individuals, lines connect individuals in the same group, and colored circles cover the individuals near the center of gravity for each group.

**Figure 5:**
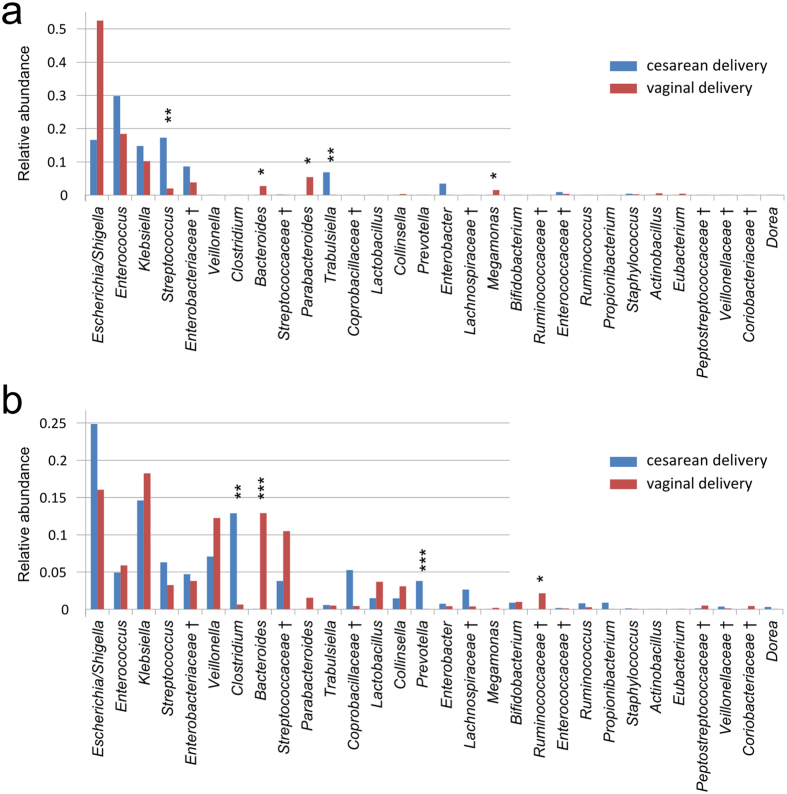
Comparison of the microbial community of vaginal and cesarean delivery subjects for neonates (**a**) and 2-month-old infants. (**b**) Only the top 30 genera are shown for clarity. Genera with significant differences between neonates and 2-month-old infants are marked by asterisks: “*” denotes *P* < 0.05; “**” denotes *P* < 0.01; “***” denotes *P* < 0.001. Unclassified genera under a higher rank are marked by “^†^”.

Despite high variability between neonates and 2-month-old infants, we were able to find discernable differences between vaginally and cesarean delivered subjects (Fig. 4a), although we detected no significant difference in the within-sample diversity and UniFrac distance. On the compositional profiles, *Bacteroidetes* was more likely to occur in vaginally delivered babies (7% vs. 0% in neonates and 13% vs. 5% in 2-month-old infants). For families within the phylum *Bacteroidetes*, *Bacteroidaceae* (mainly represented by the genera *Bacteroides* and *Parabacteroides*) tended to be enriched in vaginally delivered subjects. In contrast, *Prevotellaceae* (mainly represented by *Prevotella*) tended to be enriched in cesarean delivered subjects (Fig. 5). For other families and genera, *Streptococcus* (*p* = 0.0044, Student’s t test) and *Trabulsiella* (*p* = 0.0091) were significantly enriched in cesarean delivered neonates, and *Clostridium* (*p* = 0.0032) was significantly enriched in cesarean delivered 2-month-old infants. Another genus, *Megamonas* was only found in vaginally delivered neonates (mean relative abundance = 1.4%) and 2-month-old infants (0.3%).

We also detected significant differences according to intrapartum exposure to antibiotics. Samples that did or did not have intrapartum antibiotic exposure were clearly separated based on principal coordinate analysis (PCoA) of unweighted UniFrac distances (Fig. 4b, *p* < 0.01, Student’s t test in the first two principal components). The group separation appeared to be better and cleaner than the separation based on the grouping of vaginally and cesarean delivered subjects.

### Comparison of gut microbiota composition among infants from different countries

**Figure 6:**
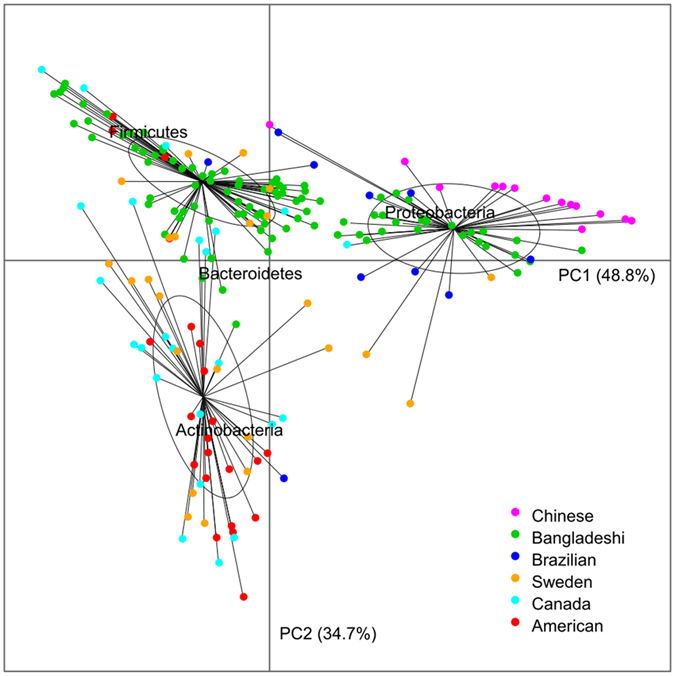
Principal component analysis of the phylum profiles in infant gut microbiota among 6 countries. The first two principle components (PCs) and their ratio of variance contribution are shown. Nodes represent the individuals, lines connect individuals in the same group, and colored circles cover the individuals near the center of gravity for each group.

A total of 206 infants from six different countries were included in this analysis (Table S1)^6^,^12^,^22^,^23^,^24^. The bacterial composition of the 206 samples was determined at each taxonomic level in individual data sets, and compared according to residence of origin. Bacterial composition at the phylum level showed marked differences among the 6 countries (Figs 6 and S1). Based on principal component analysis (PCA) of the microbial composition (Fig. 6), we separated the fecal microbiota of infants into 3 enterotypes: P-type, F-type and A-type. All the infants from China and 70% from Brazil were highly abundant in *Proteobacteria* (referred as “P-type”), whereas most of the infants in America (82%), Sweden (54%) and Canada (79%) were highly abundant in *Actinobacteria* (referred as “A-type”). 70% of the infants from Bangladeshi and 33% from Sweden were highly abundant in *Firmicutes* (referred as “F-type”). Specifically, over 45% of bacteria were *Proteobacteria* in P-type infants’ fecal samples, whereas *Proteobacteria* only accounted for 0–12.9% (average 0.7%) of gut microbiota in A-type and F-type infants.

We used permutation multivariate analysis of variance to evaluate the contributions of different factors to the variations in the infant fecal microbiota. Specifically, country of residence accounted for 19.6% (*p* = 0.0001) of the variations in infant fecal microbiota, while age, delivery mode and feed pattern accounted for only 0.42%, 0.46%, and 0.09% (*p* > 0.1) of these variations, respectively. These findings suggested that geographical/cultural related factors could have a strong and dominant impact on the composition of infant gut microbiota.

## Discussion

In this study, we applied 16S rDNA sequencing technology to characterize the gut microbiota of 29 healthy Chinese neonates and infants. In both Chinese neonates and 2-month-old infants, *Proteobacteria*, particularly *Enterobacteriaceae*, were found to be the predominant type of bacteria in the gut microbiota. More specifically, *Escherichia/Shigella* and *Klebsiella* were the main genera of *Proteobacteria* in Chinese and other P-type infants. This is in striking contrast to previous findings in Western infants^12^,^23^,^24^, for which *Actinobacteria* especially *Bifidobacterium*, was found to be the dominant genera of the intestinal microbiota.

Previous studies have also demonstrated that *Escherichia/Shigella* and *Klebsiella* were found with greater proportion in premature infants than in full-term infants^13^,^25^. *Proteobacteria* are generally considered to be potential or opportunistic pathogens due to their proinflammatory properties^26^,^27^,^28^, and the enrichment of gut *Enterobacteriaceae* is usually associated with the pathogenesis of obesity, cholecystitis and inflammatory bowel disease (IBD)^29^,^30^,^31^,^32^. In addition, early gut microbiota with a low prevalence of *Bifidobacterium adolescentis* and a high prevalence of *E. coli* and *Clostridium difficile* has been associated with allergic disease in childhood^5^. Therefore, our findings demonstrated that a relatively high abundance of harmful gut microbiota exist in P-type infants. Intriguingly, similar results have also been found when comparing the gut microbiota between Chinese and Western adults; the abundance of *Escherichia* and *Klebsiella* was over 100 fold higher, and the abundance of *Bifidobacterium* was significantly lower in Chinese compared to Danish adults^33^. Epidemiological studies have shown that atopy and asthma are more prevalent in developed and industrialized countries compared with developing countries, and early-life exposure to farming environment and farm milk has been associated with a lower risk of asthma and atopic eczema^34^. One potential interpretation of these observation is that early exposure to microbial compounds from the harmful microbes present in high abundance in P-type infants may promote earlier maturation of the immune system, thereby reducing risk for development of allergic diseases later in life.

The differences observed in within-sample diversity and UniFrac distance between vaginally and cesarean delivered infants suggested that delivery mode might affect composition of infant gut bacterial communities. *Bacteroidetes* was more abundant in the gut microbiota of vaginally delivered infants than in cesarean delivered infants, which is consistent with previous reports in Canadian^12^ and Italian infants^35^. Additionally, we found that *Clostridium* was much more abundant in the gut microbiota of cesarean delivered infants, especially at 2-months of age. On the other hand, we found that *Prevotella* was enriched in cesarean delivered Chinese infants, which is contrary to the finding of a previous study performed in Venezuela^36^ in which *Prevotella* was found to be the dominant genera in vaginally delivered infants. This discrepancy might be explained by the fact that many other major factors, besides mode of delivery, can influence composition of the gut microbiota. Other factors suggested to impact the composition of gut microbiota include feeding pattern^12^, hospitalization and prematurity^37^, antibiotic administration^35^, and the mother’s aggregate bacterial communities^36^. In fact, our further analysis showed significant differences according to intrapartum exposure to antibiotics, with much better and cleaner group separation than that based on the grouping of vaginally and cesarean delivered subjects, clearly indicating the significance of intrapartum exposure to antibiotics. In future studies, it will be interesting to comprehensively explore the association between these various factors and infant gut flora to determine the relative impact of each factor on the composition of gut microbiota.

In this work, sequences from other studies were extracted and data were re-analyzed. There is ample evidence to support the concept that experimental and technical variations could introduce bias in enterotype clustering/classification^38^,^39^. Known, conceivable, and identifiable technical differences from the various studies used for global comparison are summarized in Table 2. It is apparent that there are differences in DNA extraction methodology, type of the amplified hypervariable region, primers, and sequencing platforms in the 6 different studies. A potential limitation of our study, therefore, is that these factors may introduce some bias in our data re-analysis. However, the data (Table 2) and statistical analyses (permutation multivariate analysis), showed that samples included in the PCA in Fig. 6 do not cluster according to the different 16S variable regions or primers used. *Wesolowska-Andersen et al.*^40^ found that inter-individual variation clearly exceeded variation resulting from choice of extraction method. *Li et al.*^33^ randomly selected 11 samples to estimate bias of different DNA extraction methods (BGI vs MetaHIT); they found that metagenomes derived from the same sample using different protocols displayed high self-correlation and the same key features. Further, *Lozupone et al.*^41^ combining three studies found that culture/geography differences were large enough to outweigh technical factors, such as primers, extraction methods, and platform. In order to assess the effect of sequencing depth bias, we conducted a sensitivity analysis and set up appropriate cutoffs for low abundance OTU filtering (filtering all OTUs having an abundance lower than 3 reads) and randomly selected 1000 sequences from each sample before performing the principal coordinate analysis (See Figure S2). It appears that sequencing depth had little effect on the results. Although the potential impact of study effects cannot be ignored, we believe that the arguments presented above support the conclusion that our re-analysis of previously published data are reliable at the phyla level. Nevertheless, further investigation, including multi-center studies, are needed to confirm our initial discovery results here.

To the best of our knowledge, this is the first study to characterize the gut microbiota of healthy Chinese infants using 16s rRNA sequencing, and to compare the composition of gut microbiota in infants from different countries. Our findings have provided novel insights into our understanding of the establishment of infant fecal bacterial communities, which will help establish a framework for further studies on how altered microbiomes, early in life, influence health and disease in adolescence and adulthood.

## Additional Information

**How to cite this article**: Kuang, Y.-S. *et al.* Composition of gut microbiota in infants in China and global comparison. *Sci. Rep.* **6**, 36666; doi: 10.1038/srep36666 (2016).

**Publisher’s note:** Springer Nature remains neutral with regard to jurisdictional claims in published maps and institutional affiliations.

## Supplementary Material

Supplementary Information

## Figures and Tables

**Table 1:** Characteristics of neonates and 2-month-old infants in the study population.

Subject	Age (day)	Gender	Delivery mode	Antibiotics using
**Newborns**				
NB1	3	M	Cesarean	None
NB2	3	F	Cesarean	None
NB4	2	M	Cesarean	None
NB7	4	M	Cesarean	None
NB12	2	F	Cesarean	None
NB15	4	F	Cesarean	None
NB3	2	M	Vaginal	None
NB5	2	M	Vaginal	None
NB9	2	F	Vaginal	None
NB10	2	M	Vaginal	None
NB11	2	F	Vaginal	None
NB6	2	M	Vaginal	None
NB8	1	F	Vaginal	None
NB14	2	F	Vaginal	None
NB16	2	M	Vaginal	None
**2-month-old infants**				
TM1	73	F	Vaginal	None
TM2	43	F	Vaginal	None
TM3	44	M	Cesarean	None
TM4	34	F	Cesarean	None
TM5	70	F	Vaginal	None
TM6	41	M	Cesarean	None
TM7	91	M	Vaginal	None
TM8	49	M	Cesarean	None
TM9	42	M	Cesarean	None
TM10	50	F	Cesarean	None
TM11	42	F	Vaginal	None
TM12	66	F	Vaginal	None
TM13	71	M	Vaginal	None
TM14	44	F	Cesarean	None

**Table 2:** Relative abundance of dominant phyla in 6 different studies.

Country	Sample	DNA extraction	Average Tag/reads per sample	Platform	Technology	Microbial composition (phylum level)				
						Proteobacteria	Firmicutes	Bacteroidetes	Actinobacteria	Others
Chinese	14 samples: 1–3 month	phenol/chloroform	6,345	454	16S V3–V5	42.9	46.5	8.8	1.7	0.1
Canada^12^	24 samples: 3–4 month	FastPrep DNA for Soil Kit	8,6022	Illumina	16S V5–V7	7.4	43.8	0	36.4	12.4
Brazilian^22^	10 samples: 3 month	QIAamp DNA StoolMini-Kit	2,294	GE Healthcare	16S full length	51	29.8	0.3	9.7	9.2
Bangladeshi^6^	108 samples: 1–3 month	bead-beating in phenol/chloroform	21,218	Illumina MiSeq platform	16S V4	27.1	33.6	26.5	5.8	7
Sweden^23^	24 samples: 3 month	FastDNA SPIN Kit for Soil	2,129	Not mentioned	16S V3–V4	13.7	33.2	23.9	28.1	1.1
American^24^	22 samples: 1–3 month	bead-beating in phenol/chloroform	140,274	Illumina	16S V4	9.1	27.5	12.6	49.2	1.6
