# Supplementary material for: Composition of gut microbiota in infants in China and global comparison
Source: Sci Rep. 2016 Nov 9;6:36666. doi: 10.1038/srep36666 (PMC5101483; doi:10.1038/srep36666)
Supplement: Supplementary Information [file srep36666-s1.pdf]

Composition of gut microbiota in infants in China and global comparison

Ya-Shu Kuang[1*], Sheng-Hui Li[1*], Yong Guo[1*], Jin-Hua Lu[1], Jian-Rong He[1], Bei-Jun Luo[2], Feng-Ju Jiang[2], Hui Shen[3], Christopher J Papasian[4], Herbert Pang[5], Hui-Min Xia[1,6†], Hong-Wen Deng[3], Xiu Qiu[1,7†]

[1]Division of Birth Cohort Study, Guangzhou Women and Children's Medical Center, Guangzhou Medical University, Guangzhou 510623, People's Republic of China

[2] Department of Obstetrics, Guangzhou Women and Children's Medical Center, Guangzhou Medical University, Guangzhou 510623, People's Republic of China

[3] Center of Bioinformatics and Genomics, Department of Biostatistics and Bioinformatics, Tulane School of Public Health and Tropic Medicine, New Orleans , Louisiana ,USA

[4] Department of Basic Medical Science, School of Medicine, University of Missouri – Kansas City, Kansas City, Missouri, USA

[5]School of Public Health, Li Ka Shing Faculty of Medicine, Hong Kong, China

[6]Department of Neonatal Surgery, Guangzhou Women and Children's Medical Center, Guangzhou Medical University, Guangzhou 510623, China

[7]Department of Woman and Child Health, Guangzhou Women and Children's Medical Center, Guangzhou Medical University, Guangzhou 510623, China

[*]The authors contributed equally to this work.

[†]Correspondence to:

Xiu Qiu, Division of Birth Cohort Study, Guangzhou Women and Children's Medical Center, Guangzhou Medical University, 9 Jinsui Road, Guangzhou 510623, China; Phone: 86 2038367162; Fax: 86 2038367162; E-mail: qxiu0161@163.com.

Hui-Min Xia, Division of Birth Cohort Study, Guangzhou Women and Children's Medical Center, Guangzhou Medical University, 9 Jinsui Road, Guangzhou 510623, China; Phone: 86 2038076019; Fax: 86 2038076019; E-mail: huimin.xia876001@gmail.com.

Figure S1: Bacterial composition, at the phylum level, in infants' gut microbiota from 6 different countries. The world map was generated using R software 3.2.1 package "maps" (https://cran.r-project.org/web/packages/maps)[1]

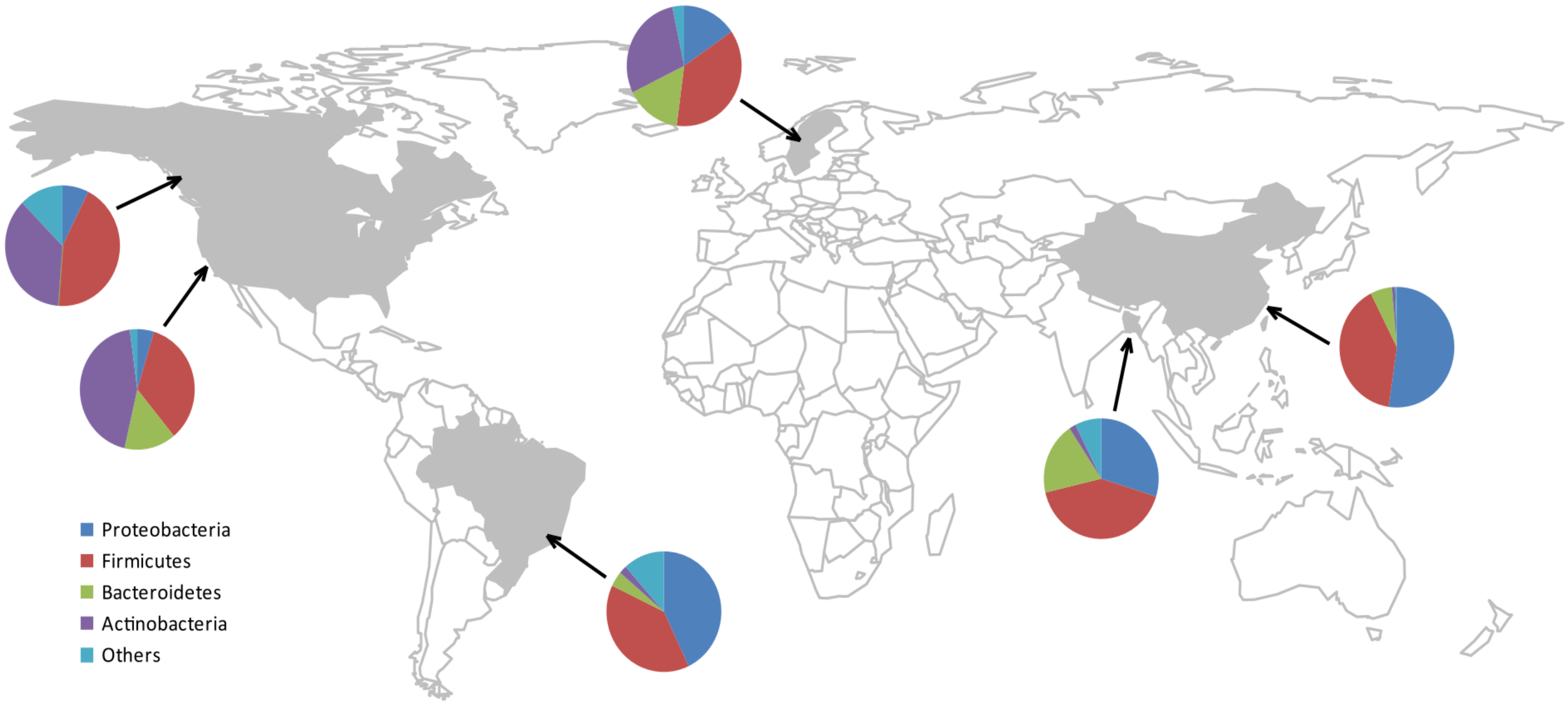

Figure S2: Effects of read depth on principal component analysis of the phylum profiles in infant gut microbiota among 6 countries. (a) (b) Taxonomic relative abundance profiles at the phylum level were also generated based on OTU annotation for each country separately. (c) (d)filtering all OTUs having an abundance lower than 3 reads.(e)(f)and randomly selected 1000 sequences from each sample.

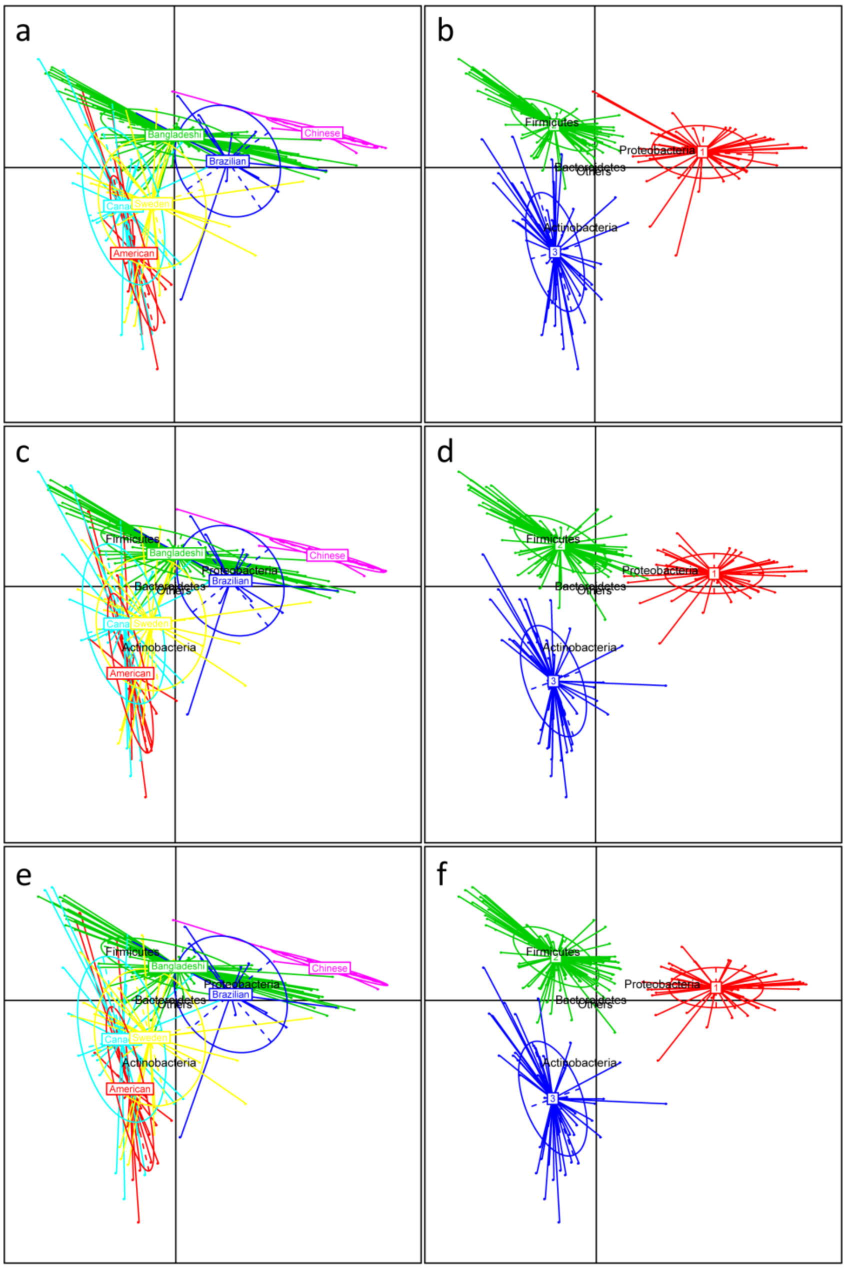

Reference

1. R Core Team (2013). R: A language and environment for statistical computing. R Foundation for Statistical Computing, Vienna, Austria. URL http://www.R-project.org/
